# Association of Electronic Nicotine Delivery System Use With Cigarette Smoking Relapse Among Former Smokers in the United States

**DOI:** 10.1001/jamanetworkopen.2020.4813

**Published:** 2020-06-05

**Authors:** Colm D. Everard, Marushka L. Silveira, Heather L. Kimmel, Daniela Marshall, Carlos Blanco, Wilson M. Compton

**Affiliations:** 1 National Institute on Drug Abuse, National Institutes of Health, Bethesda, Maryland; 2 Kelly Government Solutions, Rockville, Maryland

## Abstract

**Importance:**

Relapse to smoking among former smokers is a serious clinical concern, and use of electronic nicotine delivery systems (ENDS) has been proposed as a new risk factor for relapse. Understanding the specificity of this risk can help guide clinical practice and lead to improved health outcomes.

**Objective:**

To assess the associations of ENDS use with cigarette smoking relapse among adult former cigarette smokers.

**Design, Setting, and Participants:**

This cohort study examined data from the Population Assessment of Tobacco and Health Study, waves 1 to 4 (2013-2018). Cox proportional hazards models were developed. This is an ongoing, nationally representative, longitudinal cohort study in the US. Participants included adult (≥18 years) former cigarette smokers who reported no tobacco product use at wave 1 (unweighted n = 2273), separated into recent former cigarette smokers (last smoked ≤12 months previously) and long-term former smokers (last smoked >12 months previously). Data analysis was conducted from July to August 2019.

**Exposures:**

Self-reported use of cigarettes, ENDS, and other tobacco products (ie, cigars, pipe tobacco, hookah, snus tobacco, other smokeless tobacco, and dissolvable tobacco) was assessed.

**Main Outcomes and Measures:**

Self-reported current (every day or some days) use of cigarettes at follow-up interviews.

**Results:**

Of 2273 adult former cigarette smokers, 51.8% (95% CI, 49.7%-53.8%) were women, 65.0% (95% CI, 62.6%-67.4%) were older than 50 years, and 79.5% (95% CI, 77.8%-81.2%) were non-Hispanic white participants. Use of ENDS was associated with significant risk of cigarette smoking relapse among recent former smokers (adjusted hazard ratio [AHR], 1.63; 95% CI, 1.04-2.53; unweighted n = 304) and among long-term former smokers (AHR, 3.79; 95% CI, 1.75-8.20; unweighted n = 1554). Use of other tobacco products was also associated with significant risk for cigarette smoking relapse among recent former smokers (AHR, 1.97; 95% CI, 1.27-3.05) and among long-term former smokers (AHR, 3.82; 95% CI, 1.91-7.66).

**Conclusions and Relevance:**

In this study, use of ENDS and other tobacco products was associated with increased risk of cigarette smoking relapse among former cigarette smokers who did not use any tobacco product at wave 1 of the PATH Study. For clinicians treating former smokers who have successfully quit all nicotine products, the implications are that use of ENDS products should be discouraged, just as use of all other tobacco products is discouraged.

## Key Points

**Question:**

Is the use of electronic nicotine delivery systems associated with cigarette smoking relapse among former smokers?

**Findings:**

In a nationally representative, longitudinal cohort study in the US, use of electronic nicotine delivery systems was found to be associated with significant increases in cigarette smoking relapse for both recent former cigarette smokers (≤1 year) and long-term former cigarette smokers (>1 year).

**Meaning:**

In this study, risk of cigarette smoking relapse among former cigarette smokers was higher among those who used electronic nicotine delivery systems or other tobacco products compared with those who did not.

## Teaser

This cohort study assesses the associations of ENDS use with cigarette smoking relapse among adult former cigarette smokers.

## Introduction

Quitting cigarettes is a difficult process for most smokers, and relapse is common. Causes of relapse are multiple, and recent work suggests that use of e-cigarettes may be a risk factor for relapse to combustible cigarettes.^1,2,3^ Whether relapse is caused by the effects of a nicotine priming dose or a larger process involving nicotine triggering of a greater response to environmental cues has not been fully established.^4^ Nevertheless, the potential for use of an e-cigarette to trigger a relapse to cigarette smoking remains a major concern.

Several studies have reported other characteristics associated with relapse. García-Rodríguez et al^5^ examined a US household sample during 2 waves of data collection and concluded that the risk of relapse for former cigarette smokers was greater than 50% for those who quit within the past 12 months compared with those who had quit more than 12 months previously. That study reported that after quitting, the risk of relapse after more than 1 year decreased “hyperbolically as a function of time.”^5^ It also found that a younger age of cessation was associated with an increase in risk of relapse. Other characteristics that have been associated with relapse include having poor health, having lower socioeconomic status, having higher body mass index, being unmarried, having higher nicotine dependence, starting daily smoking at a younger age, having previous quit attempts, and having psychiatric symptoms present.^5^ Herd et al^6^ reported higher levels of relapse with lower abstinence self-efficacy (ie, belief in one’s own ability to abstain), higher frequency of smoking urges, and having a higher number of smokers among 5 closest friends.

The aim of the current study was to use the Population Assessment of Tobacco and Health (PATH) Study data to assess the association of electronic nicotine delivery systems (ENDS) use with cigarette smoking relapse among former cigarette smokers who, at wave 1, did not use any tobacco product, including ENDS. Unlike the Food and Drug Administration–approved nicotine replacement therapies, newer ENDS designs have been reported to have improved efficiency in delivering nicotine and give what are called throat-hit experiences (ie, the sensation in the throat felt during use,^7^ similar to that of smoking cigarettes^2,8,9,10^); therefore, it is of interest to assess whether the use of these products is associated with cigarette smoking relapse. Addressing gaps in previous research, the current study uses nationally representative longitudinal data collected by the PATH Study from 2013 to 2018 among US adults, which allows the inclusion of newer ENDS products. Assessing associations between ENDS use and cigarette smoking relapse among former smokers contributes to the evidence base to inform the Food and Drug Administration’s regulatory mission and to inform clinicians, public health workers, or others treating former smokers. In addition, recent (≤12 months) and long-term (>12 months) former cigarette smokers at wave 1 were assessed both together and separately to examine whether ENDS have a differential effect on those with early or established abstinence.

## Methods

### Data

Data examined in this study are from the PATH Study Restricted-Use Files. The PATH Study is an ongoing, nationally representative, longitudinal cohort study of adults and youths in the US. Self-reported information on tobacco-use behaviors was collected with audio computer-assisted self-interviews.^11^ The study used stratified, address-based, area-probability sampling design at wave 1 that oversampled adult tobacco users, young adults (aged 18-24 years), and black adults. In this study, only wave 1 cohort participants who were adults at wave 1 were studied. Full-sample and replicate weights were used that adjusted for the complex sample design and nonresponse. The weights allowed analyses of the PATH Study data to obtain statistically valid estimates for the adult, civilian, noninstitutionalized population of the US, and the replicate weights enabled computation of associated measures of statistical precision. Wave 4 all-waves weights were used to obtain statistically valid estimates from longitudinal analyses that examined wave 1 cohort data across all waves; therefore, study participants who did not respond at any of the 4 waves studied here were omitted from analysis. Further details regarding the PATH Study design and methods are published elsewhere.^11^ Details on interviewing procedures, questionnaires, sampling, weighting, response rates, and accessing the data are described in the PATH Study Restricted-Use Files user guide.^12^ The PATH Study was conducted by Westat and approved by the Westat institutional review board. All respondents provided written informed consent. This report follows the Strengthening the Reporting of Observational Studies in Epidemiology (STROBE) reporting guideline for cohort studies.^13^ Interview data were collected annually from each respondent during wave 1 (September 2013 to December 2014), wave 2 (October 2014 to October 2015), wave 3 (October 2015 to October 2016), and wave 4 (December 2016 to January 2018). Follow-up data collection protocols attempted to capture interview data from each study participant near the first anniversary of their participation in the previous wave.^12^

Current established cigarette users are defined as those who have ever smoked a cigarette, have smoked 100 cigarettes or more in their lifetime, and currently smoke every day or some days, whereas current established users of other tobacco products are those who currently use them every day or some days. Table 1 presents the variable definitions used in this study. In accordance with the previous finding that 12 months of abstinence is an important threshold for predicting relapse,^5,14^ the 3 population groups of interest examined in this study were as follows: former indicates former established cigarette smokers who did not use any tobacco product at their wave 1 interview; recent former indicates former established cigarette smokers who quit within the past 12 months of their wave 1 interview and were not using any tobacco product at that interview; and long-term former indicates former established cigarette smokers who quit more than 12 months before their wave 1 interview and were not using any tobacco product at that interview (Table 1).

**Table 1.** Population Assessment of Tobacco and Health Study Variable Descriptions

Short description	Long description, categories, or both
Current tobacco product user	Respondents who are current established cigarette smokers (ie, have ever smoked a cigarette, have smoked ≥100 cigarettes in lifetime, and currently smoke every day or some days) or currently use any other tobacco product every day or some days
Former established cigarette smoker	Respondents who have ever smoked a cigarette or have smoked ≥100 cigarettes in lifetime and now do not smoke at all
Recent former established cigarette smoker	Respondents who have ever smoked a cigarette, have smoked ≥100 cigarettes in lifetime, now do not smoke at all, and last smoked within the past 12 mo
Long-term former established cigarette smoker	Respondents who have ever smoked a cigarette, have smoked ≥100 cigarettes in lifetime, now do not smoke at all, and last smoked more than 12 mo ago
Cigarette smoking relapse	Respondents who currently smoke cigarettes every day or some days
Past 12-mo use of ENDS	Past 12-mo use of electronic nicotine products or delivery systems
Past 12-mo use of OTP	Past 12-mo use of traditional cigar, cigarillo, filtered cigar, pipe filled with tobacco, hookah, smokeless tobacco, snus, or dissolvable tobacco
Past 12-mo GAIN-SS internalizing problems	Categories: low, moderate, or high
Past 12-mo GAIN-SS externalizing problems	Categories: low, moderate, or high
Past 12-mo GAIN-SS substance use problems	Categories: low, moderate, or high
Days quit cigarettes	No. of days since last smoked cigarettes
Years quit cigarettes	No. of years since last smoked cigarettes (ie, No. of days since last smoked cigarettes divided by 365.25 and rounded to the nearest unit)
Age at which began smoking cigarettes fairly regularly	If exact age was not reported, age is set at either the midpoint of reported age range for regular smoking or current age, whichever is less
Mean cigarettes smoked/d, No.	Mean No. of cigarettes smoked per day while smoking fairly regularly
Years smoked ≥100 cigarettes, No.	Approximation of the No. of years smoked ≥100 cigarettes
Sex^a^	Categories: men or women
Age^a^	Age in years
Race/ethnicity^a^	Categories: non-Hispanic white, non-Hispanic black, non-Hispanic other race including multiracial, or Hispanic
Educational attainment^a^	Categories: <high school or GED, high school graduate, some college (no degree) or associate’s degree, bachelor’s degree, or advanced degree
Household income, $	Total household income in the past 12 mo; categories: <25 000, 25 000-49 999, 50 000-74 999, and ≥75 000

Abbreviations: ENDS, electronic nicotine delivery systems; GAIN-SS, Global Appraisal of Individual Needs–Short Screener; GED, general equivalency diploma; OTP, other tobacco products.

^a^ May contain imputed values.^12^

In these group definitions, any tobacco product refers to the tobacco products assessed in the PATH Study (ie, cigarettes, ENDS, traditional cigars, cigarillos, filtered cigars, hookah, pipe tobacco, snus tobacco, other smokeless tobacco, and dissolvable tobacco) (Table 1). During the period studied, ENDS design evolved,^15^ and consistent with this, the PATH Study questionnaire asked about e-cigarettes at wave 1 (2013-2014) and e-products (ie, e-cigarettes, e-cigars, e-pipes, and e-hookah) at waves 2, 3, and 4 (2014-2018). In the current study, we treat both e-cigarettes and e-products the same and refer to them as ENDS.

### Measures

Table 1 lists the variables used in this study. Further information on PATH Study variables can be obtained from the PATH Study codebooks.^12^ This study primarily examined associations between ENDS use and cigarette smoking relapse. Covariates examined were use of other tobacco products (ie, traditional cigar, cigarillo, filtered cigar, pipe tobacco, hookah, snus tobacco, other smokeless tobacco, and dissolvable tobacco); internalizing, externalizing, and substance use problems; past cigarette use behavior; and sociodemographic variables (sex, age, race/ethnicity, educational attainment, and household income). Internalizing, externalizing, and substance use problems were assessed by modified subscales of the Global Appraisal of Individual Needs–Short Screener and categorized as low (≤1 symptom), moderate (2-3 symptoms), or high (≥4 symptoms) severity.^16,17^ Missing data on age, sex, race/ethnicity, and educational attainment were imputed as described in the PATH Study Restricted-Use Files user guide.^12^

### Statistical Analysis

Weighted percentages with 95% CIs for sample characteristics of the 3 population groups of interest were calculated with the survey package in R version 3.5.1 (R Project for Statistical Computing). Cox proportional hazards models were developed with proc surveyphreg^18,19,20^ in SAS version 9.4 (SAS Institute). Ties were handled with the SAS TIES = EFRON option.^19^ Univariate models of time to relapse were developed for time-invariant variables determined at wave 1 (ie, for the past cigarette smoking behavior variables [time since quitting, age at initiation of regular use, cigarettes smoked per day when regularly used, and number of years of regular use] and for the sociodemographic variables [sex, age, race/ethnicity, educational attainment, and household income]). The proportional hazards assumption was checked for each time-invariant covariate assessed. Univariate models were also developed for the time-dependent variables of past 12-month use of ENDS, past 12-month use of other tobacco products, and past 12-month Global Appraisal of Individual Needs–Short Screener internalizing, externalizing, and substance use problems. To assess associations between past 12-month ENDS use and cigarette smoking relapse, Cox proportional hazards models adjusted for past 12-month other tobacco products use; past 12-month Global Appraisal of Individual Needs–Short Screener internalizing, externalizing, and substance use problems; days or years since completely quitting smoking cigarettes; sex; age; race/ethnicity; educational attainment; and household income. Statistical tests were 2-sided with significance level set at *P* < .05. Data analysis was conducted from July to August 2019.

## Results

Of adult former cigarette smokers, 51.8% (95% CI, 49.7%-53.8%) were women, 65.0% (95% CI, 62.6%-67.4%) were older than 50 years, and 79.5% (95% CI, 77.8%-81.2%) were non-Hispanic white participants. Weighted proportions for sample characteristics for all former, recent former, and long-term former groups are presented in Table 2. Within the recent former group (unweighted n = 384), there was an even distribution among the groups aged 18-30 years (31.9%; 95% CI, 25.8%-38.8%), 31-50 years (33.9%; 95% CI, 28.2%-40.2%), and older than 50 years (34.2%; 95% CI, 27.0%-42.1%), whereas within the long-term former group (unweighted n = 1886), 67.8% (95% CI, 65.2%-70.3%) were older than 50 years. The long-term former group had a higher percentage of non-Hispanic white participants (80.3%; 95% CI, 78.5%-82.1%) and a lower percentage of Hispanic participants (8.9%; 95% CI, 7.7%-10.2%) compared with the recent former group (70.7% [95% CI, 64.3%-76.4%] and 15.3% [95% CI, 10.8%-21.1%], respectively). Within the recent former group, 39.2% (95% CI, 32.1%-46.8%) had quit smoking cigarettes within the previous 122 days, whereas among the long-term former group, 14.0% (95% CI, 12.3%-15.9%) had quit cigarettes within the previous 5 years.

**Table 2.** Past Cigarette Use Behavior and Sociodemographic Characteristics at Wave 1^a^

Characteristic category^b^	Former smokers, weighted % (95% CI)^c^^,^^d^		
	All	Recent	Long-term
Unweighted, No.	2273	384	1886
Days quit cigarettes			
≤122	NA	39.2 (32.1-46.8)	NA
>122 to ≤244	NA	20.2 (15.2-26.4)	NA
>244 to ≤365	NA	40.6 (33.7-47.8)	NA
Years quit cigarettes			
≤5	21.1 (19.3-23.1)	NA	14.0 (12.3-15.9)
6-10	13.9 (12.2-15.7)	NA	15.1 (13.4-17.1)
≥11	65.0 (62.3-67.6)	NA	70.9 (68.0-73.6)
Age at which began smoking cigarettes fairly regularly, y			
<18	46.8 (43.6-50.1)	43.8 (36.3-51.5)	47.1 (43.6-50.7)
18-24	46.6 (43.7-49.6)	44.1 (36.7-51.7)	46.9 (43.6-50.1)
>24	6.5 (5.1-8.3)	12.1 (7.4-19.3)	6.0 (4.6-7.8)
Mean cigarettes smoked per day, No.			
≤10	37.4 (35.0-39.9)	51.9 (43.8-60.0)	36.0 (33.3-38.8)
11-20	40.9 (38.1-43.8)	35.6 (29.0-42.8)	41.4 (38.4-44.4)
>20	21.7 (19.3-24.4)	12.5 (7.7-19.5)	22.6 (20.0-25.4)
Years smoked ≥100 cigarettes, No.			
≤10	26.3 (24.2-28.5)	22.3 (16.5-29.4)	26.6 (24.4-29.0)
>10-≤20	29.1 (26.9-31.5)	26.5 (21.6-32.0)	29.4 (26.8-32.1)
>20	44.6 (42.3-46.9)	51.2 (43.4-59.0)	44.0 (41.4-46.5)
Sex			
Men	48.2 (46.2-50.3)	40.4 (33.5-47.6)	49.0 (46.8-51.2)
Women	51.8 (49.7-53.8)	59.6 (52.4-66.5)	51.0 (48.8-53.3)
Age, y			
18-30	6.5 (5.8-7.3)	31.9 (25.8-38.8)	4.2 (3.5-5.0)
31-50	28.5 (26.2-30.9)	33.9 (28.2-40.2)	28.0 (25.6-30.6)
>50	65.0 (62.6-67.4)	34.2 (27.0-42.1)	67.8 (65.2-70.3)
Race/ethnicity			
Non-Hispanic white	79.5 (77.8-81.2)	70.7 (64.3-76.4)	80.3 (78.5-82.1)
Non-Hispanic black	6.1 (5.1-7.3)	7.6 (4.3-12.9)	5.9 (4.9-7.2)
Non-Hispanic other, including multiracial	5.0 (4.0-6.3)	6.5 (4.1-10.2)	4.8 (3.7-6.3)
Hispanic	9.4 (8.3-10.6)	15.3 (10.8-21.1)	8.9 (7.7-10.2)
Educational attainment			
<High school or GED	13.5 (11.9-15.2)	18.1 (13.1-24.6)	13.1 (11.4-14.9)
High school graduate	24.2 (22.0-26.6)	28.0 (20.8-36.7)	23.9 (21.6-26.3)
Some college or associate’s degree	31.6 (29.7-33.6)	34.7 (28.9-41.1)	31.3 (29.3-33.4)
Bachelor’s degree	20.4 (18.5-22.5)	16.0 (11.2-22.3)	20.8 (18.8-23.0)
Advanced degree	10.3 (9.0-11.8)	3.2 (1.8-5.5)	10.9 (9.6-12.5)
Household income, $			
<25 000	24.5 (22.5-26.7)	40.2 (32.7-48.1)	23.1 (20.9-25.5)
25 000-49 999	24.4 (22.1-26.8)	23.6 (18.1-30.2)	24.5 (22.1-27.0)
50 000-74 999	18.0 (15.8-20.5)	14.3 (9.8-20.4)	18.4 (15.9-21.2)
≥75 000	33.0 (30.2-36.0)	21.9 (16.0-29.3)	34.0 (31.1-37.2)

Abbreviations: GED, general equivalency diploma; NA, not applicable to this study.

^a^ The Population Assessment of Tobacco and Health Study oversampled adult tobacco users, young adults (18-24 years), and black respondents, and weighted estimates were calculated to reflect the US civilian noninstitutionalized population at wave 1.

^b^ See Table 1 for characteristic variable descriptions.

^c^ All former indicates former established cigarette smokers who were not current users of any tobacco product at their wave 1 interview; recent former indicates former established cigarette smokers who became former cigarette smokers within the past 12 months of their wave 1 interview and were not current users of any tobacco product at that interview; and long-term former indicates former established cigarette smokers who became former cigarette smokers more than 12 months before their wave 1 interview and were not current users of any tobacco product at that interview.

^d^ 95% CIs were calculated by using the R *svyciprop* command, which computes a Wald-type interval by the logit method.

Table 3 presents univariate Cox proportional hazards analyses for these 3 groups. More detailed results from these analyses, including parameter estimates, standard errors, and *t* values, are presented in eTables 1, 2, and 3 in the Supplement. ENDS use (hazard ratio [HR], 10.01; 95% CI, 7.07-14.15); other tobacco products use (HR, 5.56; 95% CI, 4.00-7.74); past 12-month Global Appraisal of Individual Needs–Short Screener internalizing (HR, 1.38; 95% CI, 1.15-1.66), externalizing (HR, 1.23; 95% CI, 1.00-1.51), and substance use problems (HR, 1.47; 95% CI, 1.10-1.95); and time since quitting cigarettes (HR, 0.85; 95% CI, 0.81-0.89) were significantly associated with shorter time to cigarette smoking relapse.

**Table 3.** Univariate Cox Proportional Hazards Models Assessing Associations of Characteristics With Cigarette Smoking Relapse^a^

Characteristic category^b^	All former^c^		Recent former^c^		Long-term former^c^	
	Unweighted No.	HR (95% CI)	Unweighted No.	HR (95% CI)	Unweighted No.	HR (95% CI)
Past 12 mo^d^						
Use of ENDS	2223	10.01 (7.07-14.15)^e^	363	2.06 (1.34-3.19)^e^	1858	10.87 (6.54-18.08)^e^
Use of OTP	2263	5.56 (4.00-7.74)^e^	382	1.78 (1.15-2.76)^e^	1878	6.72 (3.86-11.69)^e^
GAIN-SS internalizing problems	2221	1.38 (1.15-1.66)^e^	372	1.16 (0.93-1.46)	1846	1.20 (0.91-1.58)
GAIN-SS externalizing problems	2179	1.23 (1.00-1.51)^e^	373	0.98 (0.74-1.29)	1803	1.28 (0.89-1.84)
GAIN-SS substance use problems	2150	1.47 (1.10-1.95)^e^	363	0.93 (0.64-1.34)	1784	1.47 (0.89-2.41)
Days quit cigarettes	NA	NA	384	0.998 (0.997-0.999)^e^	NA	NA
Years quit cigarettes	2270	0.85 (0.81-0.89)^e^	NA	NA	1886	0.90 (0.87-0.93)^e^
Age began smoking cigarettes fairly regularly	2024	1.02 (0.98-1.08)	351	0.99 (0.95-1.03)	1671	1.01 (0.89-1.15)
Mean cigarettes smoked/d, No.	2017	0.99 (0.95-1.04)	350	0.99 (0.96-1.02)	1667	1.00 (0.95-1.05)
Years smoked ≥100 cigarettes, No.	2259	1.01 (1.00-1.02)	384	1.00 (0.99-1.01)	1875	1.00 (0.98-1.03)
Sex						
Men	2273	1 [Reference]	384	1 [Reference]	1886	1 [Reference]
Women		1.60 (1.17-2.18)^e^		1.57 (1.08-2.30)^e^		1.40 (0.88-2.24)
Age	2273	0.95 (0.94-0.96)^e^	384	1.00 (0.98-1.01)	1886	0.95 (0.93-0.97)^e^
Race/ethnicity						
Non-Hispanic white	2273	1 [Reference]	384	1 [Reference]	1886	1 [Reference]
Non-Hispanic black		1.51 (0.91-2.50)		1.46 (0.79-2.69)		1.25 (0.53-2.96)
Non-Hispanic other race, including multiracial		0.71 (0.31-1.63)		0.56 (0.2-1.55)		0.52 (0.13-2.09)
Hispanic		1.91 (1.21-3.02)^e^		0.98 (0.51-1.91)		2.12 (1.08-4.15)^e^
Educational attainment						
<High school or GED	2273	1 [Reference]	384	1 [Reference]	1886	1 [Reference]
High school graduate		0.59 (0.33-1.05)		0.76 (0.35-1.64)		0.43 (0.17-1.11)
Some college (no degree) or associate’s degree		0.69 (0.43-1.11)		0.80 (0.44-1.44)		0.63 (0.26-1.52)
Bachelor’s degree		0.63 (0.34-1.18)		0.70 (0.31-1.59)		0.77 (0.30-1.97)
Advanced degree		0.34 (0.15-0.80)^e^		0.42 (0.13-1.40)		0.55 (0.18-1.66)
Household income, $						
<25 000	2085	1 [Reference]	350	1 [Reference]	1733	1 [Reference]
25 000-49 999		0.80 (0.51-1.27)		1.21 (0.66-2.22)		0.93 (0.45-1.91)
50 000-74 999		0.60 (0.37-0.98)^e^		0.89 (0.44-1.82)		0.74 (0.35-1.59)
≥75 000		0.51 (0.32-0.80)^e^		1.18 (0.70-1.98)		0.52 (0.25-1.05)

Abbreviations: ENDS, electronic nicotine delivery systems; GAIN-SS, Global Appraisal of Individual Needs–Short Screener; GED, general equivalency diploma; HR, hazard ratio; NA, not applicable; OTP, other tobacco products.

^a^ eTables 1, 2, and 3 in the Supplement detail these results.

^b^ See Table 1 for characteristic variable descriptions.

^c^ All former indicates former established cigarette smokers who were not current users of any tobacco product at their wave 1 interview; recent former indicates former established cigarette smokers who became former cigarette smokers within the past 12 months of their wave 1 interview and were not current users of any tobacco product at that interview; and long-term former indicates former established cigarette smokers who became former cigarette smokers more than 12 months before their wave 1 interview and were not current users of any tobacco product at that interview.

^d^ Time-dependent variables.

^e^ Significant at *P* < .05.

The Figure shows the Kaplan-Meier survival curve for relapse to cigarette smoking for the recent former and long-term former groups. The recent former group had a greater probability of relapse during the period studied than the long-term former group. There was a significant difference in survival probabilities between the 2 groups, with 95.57% for the long-term former group and 49.21% for the recent former group (*P* < .001 for the log-rank test).

**Figure.**
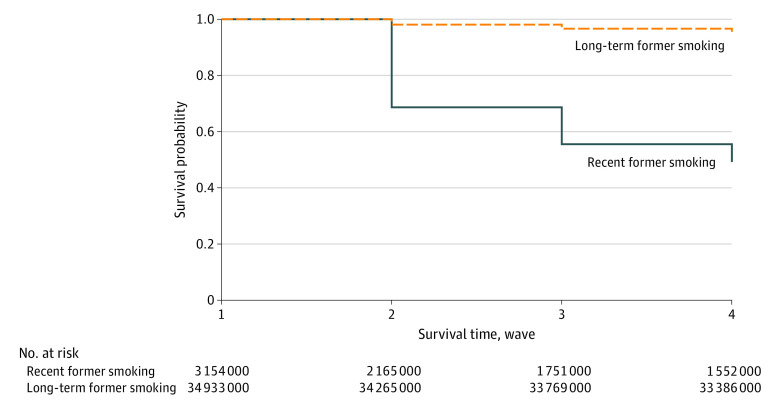
Kaplan-Meier Survival Curves of Cigarette Smoking Relapse for Recent Former Smokers and Long-term Former Smokers Numbers at risk are weighted and rounded to the nearest thousand.

Cox proportional hazards models for the 3 groups are shown in Table 4. Covariates of interest from subject matter knowledge and that were significant (ie, *P* < .05) in univariate Cox proportional hazards analyses for the all former user group were adjusted for in the Cox proportional hazards models in Table 4 (eTable 1 in the Supplement). Table 4 shows that the use of ENDS after wave 1 was associated with significant increases in the risk of cigarette smoking relapse, by 63% (95% CI, 4%-153%) for the recent former group and by 279% (95% CI, 75%-720%) for the long-term former group. Use of other tobacco products was also associated with significant increases in the risk of relapse to cigarette smoking, by 97% (95% CI, 27%-205%) for the recent former group and by 282% (95% CI, 91%-666%) for the long-term former group. Increased time since quitting before wave 1 resulted in significantly decreased risk of relapse for both these groups (for each day quit among recent former group: adjusted HR, 0.998; 95% CI, 0.996-1.00; for each year quit among long-term former group: adjusted HR, 0.93; 95% CI, 0.90-0.96). More details of the results from these analyses are presented in eTables 4, 5, and 6 in the Supplement.

**Table 4.** Cox Proportional Hazards Models With Covariates Assessing Associations of Characteristics With Cigarette Smoking Relapse^a^

Covariate^b^	Former smokers, AHR (95% CI)^c^		
	All	Recent	Long-term
Unweighted, No.	1858	304	1554
Past 12 mo^d^			
Use of ENDS	2.98 (1.93-4.60)^e^	1.63 (1.04-2.53)^e^	3.79 (1.75-8.2)^e^
Use of OTP	2.74 (1.86-4.04)^e^	1.97 (1.27-3.05)^e^	3.82 (1.91-7.66)^e^
GAIN-SS internalizing problems	1.08 (0.81-1.44)	1.16 (0.83-1.63)	1.02 (0.68-1.54)
GAIN-SS externalizing problems	0.89 (0.66-1.19)	1.01 (0.70-1.46)	0.90 (0.52-1.54)
GAIN-SS substance use problems	0.90 (0.63-1.29)	0.73 (0.45-1.19)	0.96 (0.53-1.76)
Days quit cigarettes	NA	0.998 (0.996-1.00)^e^	NA
Years quit cigarettes	0.87 (0.83-0.91)^e^	NA	0.93 (0.90-0.96)^e^

Abbreviations: AHR, adjusted hazard ratio; ENDS, electronic nicotine delivery systems; GAIN-SS, Global Appraisal of Individual Needs–Short Screener; NA, not applicable to this study; OTP, other tobacco products.

^a^ eTables 4, 5, and 6 in the Supplement detail these results. Sex, age, race/ethnicity, educational attainment, and household income are also adjusted for in these models.

^b^ See Table 1 for characteristic variable descriptions.

^c^ All former indicates former established cigarette smokers who were not current users of any tobacco product at their wave 1 interview; recent former indicates former established cigarette smokers who became former cigarette smokers within the past 12 months of their wave 1 interview and were not current users of any tobacco product at that interview; and long-term former indicates former established cigarette smokers who became former cigarette smokers more than 12 months before their wave 1 interview and were not current users of any tobacco product at that interview.

^d^ Time-dependent variables.

^e^ Significant at *P* < .05.

## Discussion

The PATH Study provides a large nationally representative US sample with which to assess cigarette smoking relapse from December 2013 to March 2018. The population of interest was former cigarette smokers who did not use any tobacco product at wave 1; that is, unlike other studies that assessed all former cigarette smokers (including former cigarette smokers who use other tobacco products),^2,3^ this study focused on those who used no tobacco products at wave 1. This is an important group because they have successfully quit cigarettes without continuing to use nicotine via noncigarette tobacco products and thus were not using ENDS to quit smoking or remain abstinent at wave 1. In this group of tobacco-abstinent individuals who quit smoking, results suggest an important role of ENDS (and other tobacco products) in relapse.

ENDS have evolved rapidly during the last 10 years,^21^ and some recent studies have investigated associations between ENDS use and cigarette smoking relapse. Dai and Leventhal^2^ used data from waves 1 and 2 of the PATH Study to assess year-to-year relapse among all former cigarette smokers (ie, including regular users of ENDS at wave 1). They found that previous and current regular use of e-cigarettes at wave 1 had significant associations with cigarette smoking relapse at follow-up compared with never use of e-cigarettes at wave 1 for those who had quit smoking cigarettes more than 12 months previously. That study did not find a significant difference between current occasional use and never use of e-cigarettes at wave 1 on cigarette smoking relapse at follow-up for those who had quit smoking cigarettes for more than 1 year. For the recent quit group (ie, those who quit smoking within 12 months of wave 1), the study reported no significant association of previous, current occasional, or current regular use of e-cigarettes vs never use of e-cigarettes with cigarette smoking relapse at follow-up. Liu et al^3^ reported that 11.9% (95% CI, 7.7%-13.0%) of ever users and 10.4% (95% CI, 6.0%-14.9%) of regular users of e-cigarettes reported restarting smoking as a consequence of e-cigarette use among a sample representative of the general population of Italy aged 15 years and older. McMillen et al^1^ studied former cigarette smokers who quit smoking at least 5 years before baseline and found that those who were users in the past 30 days or ever users of e-cigarettes were significantly more likely to have relapsed to cigarette smoking at approximately 1 year of follow-up. None of these previous studies examined prospectively the trajectories of individuals with abstinence from all tobacco products at baseline, as done in the current study.

There have been several recent developments in ENDS design to improve delivery of nicotine to the bloodstream of users.^15^ It is hypothesized that new nicotine exposure could increase the vulnerability of cigarette smoking relapse among former cigarette smokers who had quit all tobacco products. The pharmacologic effect of nicotine exposure during a smoking lapse has been associated with reinstating drug-seeking behavior and nicotine cravings.^22,23^ Reexposure to nicotine during ENDS initiation or reinitiation may result in similar outcomes. This study supports evidence that initiation or reinitiation of ENDS is significantly associated with cigarette use relapse. However, the mechanism by which ENDS use may lead to reestablishing or reinforcing nicotine-seeking behavior among former cigarette users was not specifically studied. Determining pharmacologic, behavioral, or some other explanation for these findings may require laboratory-based research. In addition, future studies can examine additional waves of the PATH Study data as they become available to further assess these associations, particularly as the variety of ENDS devices and liquids evolves. Former cigarette smokers who are free of tobacco product use should be made aware of the increased hazard of cigarette smoking relapse associated with ENDS use.

Within the PATH Study’s large, nationally representative sample of the US population, this study found that the risk of cigarette smoking relapse among individuals who were not using any tobacco products at wave 1 was significantly higher for those who subsequently used ENDS regardless of whether they had quit cigarettes recently or more than 1 year previously. The study found that recent former smokers had a greater probability of relapse during the period studied than did the long-term former group (Figure); however, the adjusted HR for relapse associated with ENDS use after wave 1 was lower for the recent former group (adjusted HR, 1.63; 95% CI, 1.04-2.53) compared with the long-term former group (adjusted HR, 3.79; 95% CI, 1.75-8.20). Taken together, these results suggest that those in the recent former group may have multiple reasons for relapse; therefore, the relative contribution to their relapse by ENDS use is significant but less pronounced than for those in the long-term former group. Our results, consistent with previous research, may be owing to several factors, such as higher frequency and intensity of withdrawal symptoms,^24^ decreased coping behaviors,^25^ increased sensitivity to smoking cues,^26^ and increased cravings or urges to smoke.^27^ Our findings converge with those of García-Rodríguez et al^5^ and suggest that relapse prevention support should be focused predominantly on recent quitters.

The current study removed individuals who were using ENDS at wave 1, which includes those who were using ENDS as a cigarette smoking cessation tool at wave 1. The population of interest may have included participants who used ENDS before their wave 1 interview. Therefore, the use of ENDS by study participants during the period studied here is attributed to initiation or reinitiation of ENDS after their wave 1 interview.

### Limitations

This study has limitations. A limitation of this study is that it did not assess different ENDS devices, different e-liquid nicotine levels, or frequency of ENDS use and their associations with cigarette smoking relapse. The PATH Study follow-up data collection protocols attempted to capture interview data from each study participant near the first anniversary of his or her participation in the previous wave,^12^ and more frequent follow-up times would have allowed increased granularity in time-to-event analysis.

## Conclusions

This study found that the use of ENDS and other tobacco products is associated with increased risk of cigarette smoking relapse among recent former and long-term former smokers who were free of tobacco product use. Decreased time since quitting cigarettes increased the risk of cigarette smoking relapse. In considering health effects of ENDS, the public health harm must be assessed along with any potential harm reduction of ENDS replacing combustible tobacco use. For the many clinicians treating former smokers who have successfully quit all nicotine products, the implications are that use of ENDS products should be discouraged, just as use of all other tobacco products is discouraged. Further research is needed to confirm and explicate our findings. In the meantime, caution is warranted for former smokers as we work to help them improve their long-term health outcomes.

## Notes

Supplement. **eTable 1. **Univariate Cox Proportional Hazards Models Assessing Associations of Characteristics With Cigarette Smoking Relapse for the All Former Group **eTable 2. **Univariate Cox Proportional Hazards Models Assessing Associations of Characteristics With Cigarette Smoking Relapse for Recent Former Group **eTable 3. **Univariate Cox Proportional Hazards Models Assessing Associations of Characteristics With Cigarette Smoking Relapse for Long-Term Former Group **eTable 4. **Cox Proportional Hazards Model with Covariates Assessing Associations of Characteristics With Cigarette Smoking Relapse for All Former Group **eTable 5. **Cox Proportional Hazards Model with Covariates Assessing Associations of Characteristics With Cigarette Smoking Relapse for Recent Former Group **eTable 6. **Cox Proportional Hazards Model with Covariates Assessing Associations of Characteristics With Cigarette Smoking Relapse for Long-Term Former Group
