# Supplementary material for: Association of Electronic Nicotine Delivery System Use With Cigarette Smoking Relapse Among Former Smokers in the United States
Source: JAMA Netw Open. 2020 Jun 5;3(6):e204813. doi: 10.1001/jamanetworkopen.2020.4813 (PMC7275247; doi:10.1001/jamanetworkopen.2020.4813)
Supplement: Supplement. — eTable 1. Univariate Cox Proportional Hazards Models Assessing Associations of Characteristics With Cigarette Smoking Relapse for the All Former Group eTable 2. Univariate Cox Proportional Hazards Models Assessing Associations of Characteristics With Cigarette Smoking Relapse for Recent Former Group eTable 3. Univariate Cox Proportional Hazards Models Assessing Associations of Characteristics With Cigarette Smoking Relapse for Long-Term Former Group eTable 4. Cox Proportional Hazards Model with Covariates Assessing Associations of Characteristics With Cigarette Smoking Relapse for All Former Group eTable 5. Cox Proportional Hazards Model with Covariates Assessing Associations of Characteristics With Cigarette Smoking Relapse for Recent Former Group eTable 6. Cox Proportional Hazards Model with Covariates Assessing Associations of Characteristics With Cigarette Smoking Relapse for Long-Term Former Group [file jamanetwopen-3-e204813-s001.pdf]

Supplementary Online Content

Everard CD, Silveira ML, Kimmel HL, Marshall D, Blanco C, Compton WM. Association of electronic nicotine delivery system use with cigarette smoking relapse among former smokers in the United States. *JAMA Netw Open*. 2020;3(6):e204813. doi:10.1001/jamanetworkopen.2020.4813

eTable 1. Univariate Cox Proportional Hazards Models Assessing Associations of Characteristics With Cigarette Smoking Relapse for the All Former Group

eTable 2. Univariate Cox Proportional Hazards Models Assessing Associations of Characteristics With Cigarette Smoking Relapse for Recent Former Group

eTable 3. Univariate Cox Proportional Hazards Models Assessing Associations of Characteristics With Cigarette Smoking Relapse for Long-Term Former Group

eTable 4. Cox Proportional Hazards Model with Covariates Assessing Associations of Characteristics With Cigarette Smoking Relapse for All Former Group

eTable 5. Cox Proportional Hazards Model with Covariates Assessing Associations of Characteristics With Cigarette Smoking Relapse for Recent Former Group

eTable 6. Cox Proportional Hazards Model with Covariates Assessing Associations of Characteristics With Cigarette Smoking Relapse for Long-Term Former Group

This supplementary material has been provided by the authors to give readers additional information about their work.

© 2020 Everard CD et al. *JAMA Network Open.*

eTable 1. Univariate Cox Proportional Hazards Models Assessing Associations of Characteristics With Cigarette Smoking Relapse for the All Former Group[a]

Characteristic[b] Category	Unweighted *n*	Estimate	Standard Error	t Value	P Value	HR (95% CI)[c]
Past 12-month use of ENDS[d]	2,223	2.303	0.1749	13.17	<0.0001	10.01 (7.07-14.15)
Past 12-month use of OTP[d]	2,263	1.716	0.1663	10.32	<0.0001	5.56 (4.00-7.74)
Past 12-month GAIN-SS internalizing problems[d]	2,221	0.324	0.0928	3.49	0.0007	1.38 (1.15-1.66)
Past 12-month GAIN-SS externalizing problems[d]	2,179	0.208	0.1041	2.00	0.0480	1.23 (1.00-1.51)
Past 12-month GAIN-SS substance use problems[d]	2,150	0.382	0.1427	2.68	0.0086	1.47 (1.10-1.95)
Years quit cigarettes	2,270	-0.167	0.0229	-7.30	<0.0001	0.85 (0.81-0.89)
Age at which began smoking cigarettes fairly regularly	2,024	0.024	0.0250	0.96	0.3377	1.02 (0.98-1.08)
Average number of cigarettes smoked per day	2,017	-0.007	0.0225	-0.31	0.7548	0.99 (0.95-1.04)
Number of years smoked 100+ cigarettes	2,259	0.012	0.0061	1.90	0.0604	1.01 (1.00-1.02)
Gender						
Male	2,273	Ref.	Ref.	Ref.	Ref.	Ref.
Female		0.469	0.1561	3.01	0.0033	1.60 (1.17-2.18)
Age	2,273	-0.054	0.0064	-8.49	<0.0001	0.95 (0.94-0.96)
Race/Ethnicity						
Non-Hispanic, White	2,273	Ref.	Ref.	Ref.	Ref.	Ref.
Non-Hispanic, Black		0.412	0.2547	1.62	0.1085	1.51 (0.91-2.50)
Non-Hispanic other, including multi-racial		-0.346	0.4203	-0.82	0.4118	0.71 (0.31-1.63)
Hispanic		0.648	0.2303	2.81	0.0059	1.91 (1.21-3.02)
Educational attainment						
Less than High School or GED	2,273	Ref.	Ref.	Ref.	Ref.	Ref.
High school graduate		-0.536	0.2943	-1.82	0.0713	0.59 (0.33-1.05)
Some college or associates degree		-0.369	0.2365	-1.56	0.1223	0.69 (0.43-1.11)
Bachelor degree		-0.466	0.3162	-1.47	0.1435	0.63 (0.34-1.18)
Advanced degree		-1.070	0.4252	-2.52	0.0135	0.34 (0.15-0.80)
Household income						
Less than $25,000	2,085	Ref.	Ref.	Ref.	Ref.	Ref.
$25,000 - $49,999		-0.220	0.2296	-0.96	0.3407	0.80 (0.51-1.27)
$50,000 - $74,999		-0.512	0.2491	-2.06	0.0423	0.60 (0.37-0.98)
$75,000 or more		-0.683	0.2292	-2.98	0.0036	0.51 (0.32-0.80)

[a]Key: ENDS, electronic nicotine delivery systems; OTP, other tobacco products, i.e., cigars, pipe tobacco, hookah, snus tobacco, other smokeless tobacco, and dissolvable tobacco; Ref., reference category. All Former, Former established cigarette smokers who were not current users of any

© 2020 Everard CD et al. *JAMA Network Open.*

tobacco product at their Wave 1 interview. [b]See Table 1 for characteristic variable descriptions. [c]HR, hazard ratio; 95% CI, 95% confidence intervals. [d]Time-dependent variables.

© 2020 Everard CD et al. *JAMA Network Open.*

eTable 2. Univariate Cox Proportional Hazards Models Assessing Associations of Characteristics With Cigarette Smoking Relapse for Recent Former Group[a]

Characteristic[b]<br>Category	Unweighted *n*	Estimate	Standard Error	t Value	P Value	HR (95% CI)[c]
Past 12-month use of ENDS[d]	363	0.725	0.2193	3.30	0.0013	2.06 (1.34-3.19)
Past 12-month use of OTP[d]	382	0.578	0.2210	2.61	0.0103	1.78 (1.15-2.76)
Past 12-month GAIN-SS internalizing problems[d]	372	0.152	0.1133	1.34	0.1821	1.16 (0.93-1.46)
Past 12-month GAIN-SS externalizing problems[d]	373	-0.022	0.1395	-0.16	0.8732	0.98 (0.74-1.29)
Past 12-month GAIN-SS substance use problems[d]	363	-0.077	0.1874	-0.41	0.6839	0.93 (0.64-1.34)
Days quit cigarettes	384	-0.002	0.0006	-3.16	0.0021	0.998 (0.997-0.999)
Age at which began smoking cigarettes fairly regularly	351	-0.012	0.0205	-0.61	0.5440	0.99 (0.95-1.03)
Average number of cigarettes smoked per day	350	-0.010	0.0136	-0.71	0.4771	0.99 (0.96-1.02)
No. of years smoked 100+ cigarettes	384	0.000	0.0062	0.04	0.9695	1.00 (0.99-1.01)
Gender						
Male	384	Ref.	Ref.	Ref.	Ref.	Ref.
Female		0.453	0.1909	2.37	0.0196	1.57 (1.08-2.30)
Age	384	-0.002	0.0071	-0.24	0.8098	1.00 (0.98-1.01)
Race/Ethnicity						
Non-Hispanic, White	384	Ref.	Ref.	Ref.	Ref.	Ref.
Non-Hispanic, Black		0.380	0.3076	1.23	0.2201	1.46 (0.79-2.69)
Non-Hispanic other, including multi-racial		-0.585	0.5170	-1.13	0.2605	0.56 (0.2-1.55)
Hispanic		-0.017	0.3351	-0.05	0.9602	0.98 (0.51-1.91)
Educational attainment						
Less than High School or GED	384	Ref.	Ref.	Ref.	Ref.	Ref.
High school graduate		-0.280	0.3915	-0.72	0.4761	0.76 (0.35-1.64)
Some college or associates degree		-0.224	0.2963	-0.76	0.4511	0.80 (0.44-1.440)
Bachelor's degree		-0.358	0.4149	-0.86	0.3900	0.70 (0.31-1.59)
Advanced degree		-0.866	0.6069	-1.43	0.1569	0.42 (0.13-1.40)
Household income						
Less than $25,000	350	Ref.	Ref.	Ref.	Ref.	Ref.
$25,000 - $49,999		0.188	0.3063	0.61	0.5416	1.21 (0.66-2.22)
$50,000 - $74,999		-0.116	0.3590	-0.32	0.7473	0.89 (0.44-1.82)
$75,000 or more		0.161	0.2628	0.61	0.5406	1.18 (0.70-1.98)

[a]Key: ENDS, electronic nicotine delivery systems; OTP, other tobacco products, i.e., cigars, pipe tobacco, hookah, snus tobacco, other smokeless tobacco, and dissolvable tobacco; Ref., reference category. Recent Former, Former established cigarette smokers who became former cigarette

© 2020 Everard CD et al. *JAMA Network Open.*

smokers within the past 12 months of their Wave 1 interview and were not current users of any tobacco product at their Wave 1 interview. [b]See Table 1 for characteristic variable descriptions. [c]HR, hazard ratio; 95% CI, 95% confidence intervals. [d]Time-dependent variables.

© 2020 Everard CD et al. *JAMA Network Open.*

eTable 3. Univariate Cox Proportional Hazards Models Assessing Associations of Characteristics With Cigarette Smoking Relapse for Long-Term Former Group[a]

Characteristic[b]<br>Category	Unweighted *n*	Estimate	Standard Error	t Value	P Value	HR (95% CI)[c]
Past 12-month use of ENDS[d]	1,858	2.386	0.2564	9.31	<0.0001	10.87 (6.54-18.08)
Past 12-month use of OTP[d]	1,878	1.905	0.2793	6.82	<0.0001	6.72 (3.86-11.69)
Past 12-month GAIN-SS internalizing problems[d]	1,846	0.181	0.1404	1.29	0.2013	1.20 (0.91-1.58)
Past 12-month GAIN-SS externalizing problems[d]	1,803	0.246	0.1831	1.34	0.1822	1.28 (0.89-1.84)
Past 12-month GAIN-SS substance use problems[d]	1,784	0.383	0.2500	1.53	0.1282	1.47 (0.89-2.41)
Years quit cigarettes	1,886	-0.102	0.0162	-6.31	<0.0001	0.90 (0.87-0.93)
Age at which began smoking cigarettes fairly regularly	1,671	0.013	0.0632	0.21	0.8315	1.01 (0.89-1.15)
Ave. no. of cigarettes smoked per day	1,667	0.000	0.0258	0.01	0.9925	1.00 (0.95-1.05)
No. of years smoked 100+ cigarettes	1,875	0.004	0.0114	0.31	0.7564	1.00 (0.98-1.03)
Gender						
Male	1,886	Ref.	Ref.	Ref.	Ref.	Ref.
Female		0.338	0.2367	1.43	0.1561	1.40 (0.88-2.24)
Age	1,886	-0.051	0.0114	-4.46	<0.0001	0.95 (0.93-0.97)
Race/Ethnicity						
Non-Hispanic, White	1,886	Ref.	Ref.	Ref.	Ref.	Ref.
Non-Hispanic, Black		0.224	0.4339	0.52	0.6073	1.25 (0.53-2.96)
Non-Hispanic other, including multi-racial		-0.664	0.7050	-0.94	0.3486	0.52 (0.13-2.09)
Hispanic		0.751	0.3393	2.21	0.0292	2.12 (1.08-4.15)
Educational attainment						
Less than High School or GED	1,886	Ref.	Ref.	Ref.	Ref.	Ref.
High school graduate		-0.845	0.4789	-1.76	0.0808	0.43 (0.17-1.11)
Some college or associates degree		-0.463	0.4442	-1.04	0.2995	0.63 (0.26-1.52)
Bachelor's degree		-0.264	0.4747	-0.56	0.5793	0.77 (0.30-1.97)
Advanced degree		-0.605	0.5611	-1.08	0.2833	0.55 (0.18-1.66)
Household income						
Less than $25,000	1,733	Ref.	Ref.	Ref.	Ref.	Ref.
$25,000 - $49,999		-0.074	0.3634	-0.20	0.8390	0.93 (0.45-1.91)
$50,000 - $74,999		-0.296	0.3822	-0.78	0.4400	0.74 (0.35-1.59)
$75,000 or more		-0.661	0.3585	-1.84	0.0681	0.52 (0.25-1.05)

[a]Key: ENDS, electronic nicotine delivery systems; OTP, other tobacco products, i.e., cigars, pipe tobacco, hookah, snus tobacco, other smokeless tobacco, and dissolvable tobacco; Ref., reference category. Long-Term Former, Former established cigarette smokers who became former

© 2020 Everard CD et al. *JAMA Network Open.*

cigarette smokers more than 12 months before their Wave 1 interview and who were not current users of any tobacco product at their Wave 1 interview. [b]See Table 1 for characteristic variable descriptions. [c]HR, hazard ratio; 95% CI, 95% confidence intervals. [d]Time-dependent variables.

© 2020 Everard CD et al. *JAMA Network Open.*

eTable 4. Cox Proportional Hazards Model with Covariates Assessing Associations of Characteristics With Cigarette Smoking Relapse for All Former Group

(Unweighted *n* = 1858)[a].

Covariate[b]<br>Category	Estimate	Standard Error	t Value	P Value	AHR (95% CI)[c]
Past 12-month use of ENDS[d]	1.091	0.2192	4.98	<0.0001	2.98 (1.93-4.60)
Past 12-month use of OTP[d]	1.009	0.1947	5.18	<0.0001	2.74 (1.86-4.04)
Past 12-month GAIN-SS internalizing problems[d]	0.074	0.1458	0.51	0.6122	1.08 (0.81-1.44)
Past 12-month GAIN-SS externalizing problems[d]	-0.119	0.1474	-0.81	0.4208	0.89 (0.66-1.19)
Past 12-month GAIN-SS substance use problems[d]	-0.104	0.1814	-0.58	0.5660	0.90 (0.63-1.29)
Years quit cigarettes	-0.142	0.0244	-5.81	<0.0001	0.87 (0.83-0.91)
Gender					
Male	Ref.	Ref.	Ref.	Ref.	Ref.
Female	-0.396	0.2039	-1.94	0.0550	0.67 (0.45-1.01)
Age	-0.002	0.0067	-0.37	0.7130	1.00 (0.98-1.01)
Race/Ethnicity					
Non-Hispanic, White	Ref.	Ref.	Ref.	Ref.	Ref.
Non-Hispanic, Black	-1.202	0.4891	-2.46	0.0157	0.30 (0.11-0.79)
Non-Hispanic other, including multi-racial	-0.207	0.2842	-0.73	0.4688	0.81 (0.46-1.43)
Hispanic	-0.516	0.2278	-2.27	0.0256	0.60 (0.38-0.94)
Educational attainment					
Less than High School or GED	Ref.	Ref.	Ref.	Ref.	Ref.
High school graduate	-0.251	0.2578	-0.98	0.3319	0.78 (0.47-1.30)
Some college or associates degree	-0.019	0.3903	-0.05	0.9604	0.98 (0.45-2.13)
Bachelor degree	-0.165	0.5224	-0.32	0.7526	0.85 (0.30-2.39)
Advanced degree	0.037	0.2861	0.13	0.8981	1.04 (0.59-1.83)
Household income					
Less than $25,000	Ref.	Ref.	Ref.	Ref.	Ref.
$25,000 - $49,999	-0.017	0.3188	-0.05	0.9567	0.98 (0.52-1.85)
$50,000 - $74,999	-0.134	0.3883	-0.34	0.7316	0.88 (0.41-1.89)
$75,000 or more	0.126	0.2427	0.52	0.6060	1.13 (0.70-1.84)

[a]Key: ENDS, electronic nicotine delivery systems; OTP, other tobacco products, i.e., cigars, pipe tobacco, hookah, snus tobacco, other smokeless tobacco, and dissolvable tobacco; Ref., reference category. All Former, Former established cigarette smokers who were not current users of any tobacco product at their Wave 1 interview.

© 2020 Everard CD et al. *JAMA Network Open.*

[b]See Table 1 for characteristic variable descriptions.

[c]AHR, adjusted hazard ratio; 95% CI, 95% confidence intervals.

[d]Time-dependent variables.

© 2020 Everard CD et al. *JAMA Network Open.*

eTable 5. Cox Proportional Hazards Model with Covariates Assessing Associations of Characteristics With Cigarette Smoking Relapse for Recent Former Group

(Unweighted *n* = 304)[a].

Covariate[b] Category	Estimate	Standard Error	t Value	P Value	AHR (95% CI)[c]
Past 12-month use of ENDS[d]	0.485	0.2231	2.18	0.0320	1.63 (1.04-2.53)
Past 12-month use of OTP[d]	0.677	0.2208	3.06	0.0028	1.97 (1.27-3.05)
Past 12-month GAIN-SS internalizing problems[d]	0.150	0.1715	0.87	0.3843	1.16 (0.83-1.63)
Past 12-month GAIN-SS externalizing problems[d]	0.010	0.1838	0.06	0.9556	1.01 (0.70-1.46)
Past 12-month GAIN-SS substance use problems[d]	-0.316	0.2451	-1.29	0.1996	0.73 (0.45-1.19)
Days quit cigarettes	-0.002	0.0008	-2.46	0.0155	0.998 (0.996-1.00)
Gender					
Male	Ref.	Ref.	Ref.	Ref.	Ref.
Female	-0.545	0.2796	-1.95	0.0540	0.58 (0.33-1.01)
Age	0.004	0.0077	0.46	0.6455	1.00 (0.99-1.02)
Race/Ethnicity					
Non-Hispanic, White	Ref.	Ref.	Ref.	Ref.	Ref.
Non-Hispanic, Black	-1.146	0.5578	-2.05	0.0425	0.32 (0.11-0.96)
Non-Hispanic other race including multi-racial	-0.567	0.4670	-1.21	0.2277	0.57 (0.23-1.43)
Hispanic	-0.392	0.3513	-1.12	0.2673	0.68 (0.34-1.36)
Educational attainment					
Less than High School or GED	Ref.	Ref.	Ref.	Ref.	Ref.
High school graduate	-0.339	0.2951	-1.15	0.2537	0.71 (0.40-1.28)
Some college (no degree) or associates degree	-0.403	0.4121	-0.98	0.3304	0.67 (0.30-1.51)
Bachelor's degree	-1.160	0.6707	-1.73	0.0867	0.31 (0.08-1.19)
Advanced degree	0.430	0.4164	1.03	0.3040	1.54 (0.67-3.51)
Household income					
Less than $25,000	Ref.	Ref.	Ref.	Ref.	Ref.
$25,000 - $49,999	-0.550	0.4529	-1.21	0.2276	0.58 (0.24-1.42)
$50,000 - $74,999	0.171	0.3580	0.48	0.6331	1.19 (0.58-2.42)
$75,000 or more	-0.384	0.3431	-1.12	0.2653	0.68 (0.35-1.35)

[a]Key: ENDS, electronic nicotine delivery systems; OTP, other tobacco products, i.e., cigars, pipe tobacco, hookah, snus tobacco, other smokeless tobacco, and dissolvable tobacco; Ref., reference category. Recent Former, Former established cigarette smokers who became former cigarette smokers within the past 12 months of their Wave 1 interview and were not current users of any tobacco product at their Wave 1 interview.

[b]See Table 1 for characteristic variable descriptions.

© 2020 Everard CD et al. *JAMA Network Open.*

[c]AHR, adjusted hazard ratio; 95% CI, 95% confidence intervals.

[d]Time-dependent variables.

© 2020 Everard CD et al. *JAMA Network Open.*

eTable 6. Cox Proportional Hazards Model with Covariates Assessing Associations of Characteristics With Cigarette Smoking Relapse for Long-Term Former Group (Unweighted *n* = 1554)[a].

Covariates[b]<br>Category	Estimate	Standard Error	t Value	P Value	AHR (95% CI)[c]
Past 12-month use of ENDS[d]	1.333	0.3894	3.42	0.0009	3.79 (1.75-8.20)
Past 12-month use of OTP[d]	1.341	0.3503	3.83	0.0002	3.82 (1.91-7.66)
Past 12-month GAIN-SS internalizing problems[d]	0.023	0.2065	0.11	0.9129	1.02 (0.68-1.54)
Past 12-month GAIN-SS externalizing problems[d]	-0.108	0.2732	-0.39	0.6939	0.90 (0.52-1.54)
Past 12-month GAIN-SS substance use problems[d]	-0.039	0.3054	-0.13	0.8977	0.96 (0.53-1.76)
Years quit cigarettes	-0.077	0.0168	-4.55	<0.0001	0.93 (0.90-0.96)
Gender					
Male	Ref.	Ref.	Ref.	Ref.	Ref.
Female	-0.267	0.2637	-1.01	0.3135	0.77 (0.45-1.29)
Age	-0.012	0.0112	-1.03	0.3069	0.99 (0.97-1.01)
Race/Ethnicity					
Non-Hispanic, White	Ref.	Ref.	Ref.	Ref.	Ref.
Non-Hispanic, Black	-1.353	0.7603	-1.78	0.0782	0.26 (0.06-1.17)
Non-Hispanic other, including multi-racial	-0.022	0.4903	-0.04	0.9644	0.98 (0.37-2.59)
Hispanic	-0.577	0.4318	-1.34	0.1843	0.56 (0.24-1.32)
Educational attainment					
Less than High School or GED	Ref.	Ref.	Ref.	Ref.	Ref.
High school graduate	0.079	0.4101	0.19	0.8473	1.08 (0.48-2.44)
Some college or associates degree	0.461	0.5751	0.8	0.4247	1.59 (0.51-4.96)
Bachelor degree	0.559	0.6977	0.8	0.4247	1.75 (0.44-6.98)
Advanced degree	0.069	0.4368	0.16	0.8751	1.07 (0.45-2.55)
Household income					
Less than $25,000	Ref.	Ref.	Ref.	Ref.	Ref.
$25,000 - $49,999	0.094	0.4369	0.22	0.8294	1.10 (0.46-2.62)
$50,000 - $74,999	-0.496	0.5968	-0.83	0.4082	0.61 (0.19-1.99)
$75,000 or more	0.159	0.3394	0.47	0.6409	1.17 (0.60-2.30)

[a]Key: ENDS, electronic nicotine delivery systems; OTP, other tobacco products, i.e., cigars, pipe tobacco, hookah, snus tobacco, other smokeless tobacco, and dissolvable tobacco; Ref., reference category. Long-Term Former, Former established cigarette smokers who became former cigarette smokers more than 12 months before their Wave 1 interview and who were not current users of any tobacco product at their Wave 1 interview.

[b]See Table 1 for characteristic variable descriptions.

© 2020 Everard CD et al. *JAMA Network Open.*

[c]AHR, adjusted hazard ratio; 95% CI, 95% confidence intervals. [d]Time-dependent variables.

© 2020 Everard CD et al. *JAMA Network Open.*
